# The Role of BDNF on Aging-Modulation Markers

**DOI:** 10.3390/brainsci10050285

**Published:** 2020-05-09

**Authors:** Claudio Molinari, Vera Morsanuto, Sara Ruga, Felice Notte, Mahitab Farghali, Rebecca Galla, Francesca Uberti

**Affiliations:** Laboratory of Physiology, Department of Translational Medicine, University of Piemonte Orientale, Via Solaroli 17, 28100 Novara, Italy; claudio.molinari@med.uniupo.it (C.M.); vera.morsanuto@med.uniupo.it (V.M.); sara.ruga@uniupo.it (S.R.); felice.notte@uniupo.it (F.N.); mahitab.farghali@uniupo.it (M.F.); rebecca.galla@uniupo.it (R.G.)

**Keywords:** low dose BDNF, brain aging, astrocytes, BBB, in vivo model

## Abstract

An important link between brain aging and a class of growth/survival factors called neurotrophins has recently been demonstrated. In particular, brain-derived neurotrophic factor (BDNF) plays a fundamental role during age-related synaptic loss, preventing cerebral atrophy and cognitive decline. The aim of the present study was to investigate whether the use of low dose BDNF sequentially kinetic activated (SKA) was able to counteract some mechanisms underlying the degeneration and aging of nervous tissue by increasing endogenous protection mechanisms. Both in vitro and in vivo experiments were performed to assess the ability of BDNF SKA to protect and regenerate survival-related molecular pathways, studying intestinal absorption in vitro and brain function in vivo. Our pioneering results show that BDNF SKA is able to induce the endogenous production of BDNF, using its receptor TrkB and influencing the apolipoprotein E expression. Moreover, BDNF SKA exerted effects on β-Amyloid and Sirtuin 1 proteins, confirming the hypothesis of a fine endogenous regulatory effect exerted by BDNF SKA in maintaining the health of both neurons and astrocytes. For this reason, a change in BDNF turnover is considered as a positive factor against brain aging.

## 1. Introduction

Research on brain aging and on neurodegenerative diseases is one of the most important challenges that the scientific community has been facing in recent years. The mechanisms underlying brain aging are many and closely related. Recently, an important role has been demonstrated for a class of cell growth and survival factors called neurotrophins [1]. Neurotrophins belong to a family of small proteins playing a fundamental role in both the central and the peripheral nervous systems. The main functions of these proteins are the regulation of axonal growth and neuronal differentiation. The neurotrophins family includes nerve growth factor, neurotrophin-3, neurotrophin-4/5 and, even more important, brain-derived neurotrophic factor (BDNF) [2]. BDNF is the most abundant neurotrophin in the brain and appears essential for neuronal survival during the development and formation of neural networks in the adult brain, exerting its biological actions through tyrosine receptor kinase B (TrkB receptors) [3]. It is produced in the brain by different types of cells and can be transported outside the brain through the blood–brain barrier (BBB). BDNF plays a fundamental role during brain development as it supports the survival and differentiation of neuronal populations of the peripheral and central nervous systems and regulates synaptogenesis, synaptic transmission and plasticity. Furthermore, BDNF plays a crucial role in learning and memory mechanisms [4]. BDNF and other neurotrophins are now considered to be growth factors with a wide spectrum of functions also outside the nervous system, including the modulation and regulation of the immune function [5]. Moreover, BDNF and its specific precursors may have a role in sustaining thymocyte precursor survival and supporting the thymocyte differentiation process [6].

A preclinical study stated that the dysregulation of BDNF signaling is involved in several neurodegenerative disorders, including Alzheimer’s disease, and leads to a deficit in age-related learning [7]. BDNF has also been shown to be able to interact with oxygen radicals (ROS) whose imbalance is involved in the mechanisms of aging, neurodegenerative diseases and some neuropsychiatric disorders [7]. In brain aging there is a decline in the normal antioxidant potential, which leads to an increase in the brain’s vulnerability to the harmful effects of oxidative damage [8]. BDNF is considered one of the protective agents against oxidative stress in the central nervous system [9]. Some brain areas particularly involved in neurodegenerative diseases such as the hippocampus, the *substantia nigra*, the amygdala and the frontal cortex are particularly sensitive to oxidative stress [10]. The role of ROS as a marker of brain aging is supported by numerous studies demonstrating that an increase in these substances is related to the reduction of mitochondrial function [11,12]. Several clinical studies have shown changes in blood concentration of BDNF in patients with neuropsychiatric disorders such as major depression [13], schizophrenia [14] and Alzheimer’s disease [15]. Studies conducted both in vitro and in vivo have shown that the expression of BDNF and of its specific TrkB receptor is essential to keep an appropriate number of proliferating stem cells, for the differentiation of neuronal populations and for the maturation of excitatory synapses [2].

The possibility of using exogenous BDNF as a therapeutic approach against neurodegenerative diseases has been hypothesized in recent years. However, supplementation of exogenous BDNF presents several problems. The main one depends on the amount of BDNF that reaches the brain. If such amount is too low, it might not be enough to produce the desirable effects. On the other hand, if it is too high it might paradoxically be dangerous, as it might cause, for example, TrkB receptors to be downregulated, thus reducing the intracellular machinery linked to BDNF. The possibility that BDNF can cross the BBB is rather controversial. While some authors argue that it is not clear whether BDNF can readily cross the BBB [16], others indicate that BDNF is able to [17]. In an attempt to overcome such delivery problems, different methods have been developed, which, however, did not thoroughly solve the problem [18]. Other studies have reported further problems related to the administration of neurotrophins in humans, depending on the dose and pharmacokinetics of these molecules [19]. A promising way to achieve a fine regulation of physiological mechanisms could be the use of low-dose substances, as demonstrated by numerous studies available in the literature [20,21]. Several experimental studies have also shown that the administration of low-dose bioactive molecules is effective to obtain clean biological effects with a low probability of adverse effects.

To date, there are no therapies capable of blocking the neuronal death process that triggers neurodegenerative diseases. The use of the family of neurotrophic factors BDNF is part of, whose deficiency has been found in brain aging and in various pathological conditions, could become a valid therapeutic solution. However, there are still numerous problems to be solved, such as the calculation of the dose, the protocol of administration and the crossing of the BBB. Therefore, the aim of this study was to analyze the effect of low doses of BDNF on ROS production in both cultured astrocytes and cortical neurons by observing the behavior of endogenous antioxidant mechanisms. Furthermore, experiments were carried out in order to improve the tolerability of the substance by studying its ability to exert beneficial effects on the molecular pathways linked to viability in the nervous tissue. Finally, experiments were conducted both in vitro and in vivo to evaluate the characteristics of intestinal absorption after oral intake of BDNF and to evaluate the ability of BDNF to cross the BBB.

## 2. Materials and Methods

### 2.1. Preparation of BDNF Solutions

All dilutions were prepared starting from a stock solution (0.001 ng/mL) of BDNF 4CH in 0.9% NaCl. Based on previous knowledge on activated blends [21,22], BDNF solutions were prepared at two different concentrations: 1 pg/mL for in vitro studies and 1.20 pg/mL for in vivo studies. Each concentration was prepared using the sequential kinetic activation (SKA) method [21]. These solutions are kinetically energized by a mechanically applied force via a standardized shaking process (sequential kinetic activation named SKA), characterized by vertical shaking corresponding to 100 oscillations in 10 s. All solutions were prepared by GUNA Laboratories (GUNA S.p.a, Milan, Italy). For each treatment, the volume of each solution was calculated by comparing the volume added to the sample treated with 50 ng/mL BDNF for in vitro study [23] and with 25ng/mL BDNF [24] for in vivo experiments. The BDNF used to compare the results obtained with BDNF 1 pg/mL SKA was not subjected to SKA treatment in order to replicate the same experimental conditions as in other studies.

### 2.2. Astrocytes Isolation

Primary mouse astrocyte cultures were extracted from C57BL/6 mouse pups, following a classical technique [25] according to the National Guideline for the Use and Care of Laboratory Animals. Briefly, within 24 h of birth pups were euthanized, and cortices were dissected, minced, mechanically digested and left to settle for 30 min at room temperature. The cell suspension was centrifuged at 800 rpm for 5 min and pelleted cells were resuspended in Neuronal Basal Medium (Sigma-Aldrich, Milan, Italy) supplemented with 5% fetal bovine serum (FBS, Sigma-Aldrich, Milan, Italy), 1% penicillin/streptomycin (Sigma-Aldrich, Milan, Italy) and 2 mM L-glutamine (Sigma-Aldrich, Milan, Italy), plated in multi-wells and maintained in culture for 6 days before treatment. The cells were plated 4 × 104 astrocytes/cm^2^ on a 24-well Transwell support to prepare the model BBB; 1 × 104 on 96-well plates for MTT and crystal violet staining; 4 × 104 cells were plated in black 96-well plates to study o × ygen consumption and mitochondrial membrane potential; 5 × 104 cells were plated on 24-well plates to analyze reactive o × ygen species (ROS) production; 2 × 105 cells were plated on 24-well plates to quantify BDNF; and 1 × 106 in 6-well plates to analyze the intracellular pathways by Western blot and ERK activity by ELISA test.

### 2.3. Primary Cortical Neuronal Cells

Primary mouse cortical neuronal cultures were obtained from the brains of P0 C57BL/6 mouse pups, as reported in literature [26]. All procedures used in these studies follow the guidelines in accordance with the National Institutes of Health Guidelines. Cortices were dissected from embryonic brains and the tissue was mechanically dissociated and left to settle for 30 min at room temperature. After centrifuging only, the supernatant was re-suspended in Neuronal Basal medium (Sigma-Aldrich, Milan, Italy) supplemented with 2% B27 (Sigma-Aldrich, Milan, Italy), 1% penicillin/streptomycin (Sigma-Aldrich, Milan, Italy) and 2 mM L-glutamine (Sigma-Aldrich, Milan, Italy). Cells were plated on pre-coated plates with 10 µg/mL poly-L-lysine at a density of 1 × 106 cells/mL and were maintained in incubator at 37 °C with 5% CO2 and 95% humidity. At three days from plating, the medium was changed. All experiments were performed on primary cortical neuronal cells grown for 9–10 days in vitro. The cells were plated 1 × 104 on 96-well plates for MTT; 5 × 104 cells were plated on 24-well plates to analyze reactive oxygen species (ROS) production; 2 × 105 cells were plated on 24-well plates to quantify BDNF; and 4 × 104 cells were plated in black 96-well plates to study oxygen consumption and mitochondrial membrane potential.

### 2.4. In Vitro Experimental Protocol

Before treatment, both primary cortical neuronal cells and astrocytes were maintained in Dulbecco’s modified Eagle’s medium (DMEM, Sigma-Aldrich, Milan, Italy) without red phenol and FBS, supplemented with 1% penicillin/streptomycin (Sigma-Aldrich, Milan, Italy) 2 mM L-glutamine (Sigma-Aldrich, Milan, Italy) and 1 mM sodium pyruvate (Sigma-Aldrich, Milan, Italy) at 37 °C, 5% CO2 and 95% humidity for 1 h. Cells were treated with 1 pg/mL BDNF SKA and 50 ng/mL BDNF at T0, checked every 24 h, and maintained for 6 days (named 6 days protocol). The vehicle, a saline solution, was also analyzed. Moreover, the involvement of TrkB using a specific antagonist, 1 µg/mL ANA-12 (Sigma-Aldrich, Milan, Italy) [27] treating cells 30 min before stimulation was investigated. Additional experiments were carried out to analyze the ability of BDNF solutions to restore the damage caused by oxidative stress, a major cause of aging and neurodegeneration. Both cortical neuronal cells and astrocytes were pre-treated with 200 μM H_2_O_2_ (Sigma-Aldrich, Milan, Italy) [28] for 30 min and then treated with 1 pg/mL BDNF SKA and 50 ng/mL BDNF. Finally, astrocytes were used in time-course study within 24 h to mimic the human posology.

### 2.5. Intestinal Barrier In Vitro Model

Caco-2 cells (human epithelial colorectal adenocarcinoma cells), purchased from American Type Culture Collection (ATCC, Manassas, VA, USA), were used as an experimental model [29] to predict the features of intestinal absorption following oral intake [30]. These cells were grown in a complete medium composed of Dulbecco’s Modified Eagle’s Medium/Nutrient F-12 Ham (DMEM-F12, Sigma-Aldrich, Milan, Italy) supplemented with 10% fetal bovine serum (FBS, Sigma-Aldrich, Milan, Italy), 2 mM L-glutamine (Sigma-Aldrich, Milan, Italy), 1% penicillin-streptomycin (Sigma-Aldrich, Milan, Italy) and maintained in an incubator at 37 °C with 5% CO2 and 95% humidity. Cells were used from passages 46 to 49 and seeded in 24-well polyester Corning^®^ Costar^®^ transwell plates (Sigma-Aldrich, Milan, Italy) in complete medium. The cells were cultured for up to 21 days in a humidified incubator maintained at 37 °C in an atmosphere of 5% CO2, changing medium every 3 days first basolaterally and then apically. The monolayer integrity was checked every 3 days (at the time of the medium change) [31]. After 21 days, 1pg/mL BDNF SKA and 50 ng/mL BDNF were added to culture medium under different pH conditions, as reported in literature [31]; pH 6.5 preparations were added to the apical side, whereas pH 7.4 was added to the basolateral side. The slightly acidic pH (pH 6.5) in the apical side represents the average pH in the lumen of the small intestine, whereas the neutral pH (pH 7.4) in the basolateral side mimics the pH of the blood. During treatments, the cells were maintained in an incubator at 5% CO2, and at the end of stimulations the BDNF quantity was measured by ELISA kit after 30 min and 1, 3, 4, 5and 6h from stimulation. This model is suitable to predict the absorption of substances after oral intake by evaluating the apparent permeability coefficient (Papp) [31,32]. Briefly, the Papp (cm/s) was calculated as [29]:

Papp = dQ/dt × 1/m0 × 1/A × VDonor

dQ: amount of substance transported (nmol or μg)

dt: incubation time (sec)

m0: amount of substrate applied to donor compartment (nmol or μg)

A: surface area of transwell membrane (cm^2^)

VDonor: volume of the donor compartment (cm^3^)

Negative controls without cells were tested to exclude transwell membranes influence.

### 2.6. Blood–Brain Barrier (BBB) Experimental Model

Astrocytes were co-cultured with human umbilical vein endothelial cells (HUVEC) cells according to methods reported in literature [33]. HUVEC were purchased from ATCC^®^. Cells were cultured in EGM Media (Lonza, Basel, Switzerland) supplemented with 10% FBS (Sigma-Aldrich, Milan, Italy), 1% penicillin/streptomycin (Sigma-Aldrich, Milan, Italy) and 2 mM Glutamine (Sigma-Aldrich, Milan, Italy) at 37 °C in a humidified atmosphere of 95% air, 5% CO2. In brief to create the BBB barrier, 4 × 104 astrocytes/cm^2^ were plated on the basolateral side of the flipped 6.5 mm Transwells with polyester membrane with 0.4 μm pore size (Corning Costar, Sigma-Aldrich, Milan, Italy) and left to attach for 4 h. Transwells were then placed into the normal orientation and the cells left to grow for 48 h. After this time, 1 × 105 HUVEC cells/cm^2^ were plated in the apical compartment. The inserts were then placed in a 24-well plate. After 7 days of culture, the Transwells were treated and permeability studies were performed [34]. To understand the ability of tested substances to cross the blood–brain barrier the medium at the bottom side of the Transwells was quantified over time by measuring the volume and the concentration of BDNF.

### 2.7. Brain-Derived Neurotrophic Factor (BDNF) Quantification

Brain-derived neurotrophic factor (BDNF) quantification was measured by Rat BDNF Elisa Kit (Thermo ScientificTM, Waltham, MA, United States) in cellular supernatants obtained from basolateral environment of BBB, primary cortical neuronal cells, astrocytes, serum and brain tissue to quantify BDNF, following the manufacturer’s instructions. Tissues were washed in saline solution, weighed, cut in small pieces and homogenized 100 mg tissue/300  μL with cold lysis buffer (0.1  M Tris, 0.01  M NaCl, 0.025  M EDTA, 1% NP40, 1% Triton X-100; Sigma-Aldrich, Milan, Italy) supplemented with 2 mM sodium orthovanadate, 0.1 M sodium fluoride (Sigma-Aldrich, Milan, Italy), 1:100 mix of protease inhibitors (Sigma-Aldrich, Milan, Italy), and 1:1000 phenylmethylsulfonyl fluoride (PMSF; Sigma-Aldrich, Milan, Italy), using an electric potter at 1600  rpm for 2  min. The tissue extracts were centrifuged at 13000  rpm for 20  min at 4 °C.

The cellular and tissue supernatants were collected, and each sample was tested by ELISA kit. Briefly, biotinylated detection antibody was added into each well and the plate was incubated for 1 h at room temperature. Then, after 45 min of incubation with HRP-conjugated streptavidin, TMB substrate solution was added for 30 min and subsequently the reaction was stopped by adding Stop Solution. BDNF concentration was determined by measuring the absorbance through a spectrometer (VICTOR X4, multilabel plate reader) at 450  nm and calculated by comparing results to BDNF standard curve.

### 2.8. MTT Assay

MTT-based In Vitro Toxicology Assay Kit (Sigma-Aldrich, Milan, Italy) was performed on both cell types to determine cell viability, as previously described [35]. Briefly, at the end of each stimulation, the cells were incubated with 1% MTT dye for 2 –3 h at 37 °C in incubator, until the purple crystals were dissolved in equal volume of MTT Solubilization Solution. The relative viability (%) was based on absorbance measuring through a spectrometer (VICTOR X4, Multilabel Plate Reader) at 570 nm with correction at 690 nm. Finally, viability was calculated comparing results to control cells (defined as 100% viable).

### 2.9. Crystal Violet Staining

After each treatment astrocytes were fixed with 1% glutaraldehyde (Sigma-Aldrich, Milan, Italy) for 15 min at room temperature, washed, and stained with 100 µL 0.1% aqueous crystal violet (Sigma-Aldrich, Milan, Italy) for 20 min at room temperature. To multi-well plates, 100 µL of 10% acetic acid was added and mixed before reading the absorbance at 595 nm using a spectrometer (VICTOR X4, multilabel plate reader). The estimated number was calculated by comparing the results to the control cells counted at T0.

### 2.10. ROS Production

The rate of reactive species of oxygen (ROS) was measured using a standard protocol based on the addition of cytochrome C (Sigma-Aldrich, Milan, Italy) to the samples and to another sample of 100 μL superoxide dismutase (Sigma-Aldrich, Milan, Italy). They were added for 30 min in an incubator at 37 °C, 5% CO_2_, and 95% humidity to determine the antioxidant capability of BDNF solutions on the BBB model, primary cortical neuronal cells and astrocytes. At the end of stimulations, 100 µL of supernatant were measured at 550 nm using a spectrometer (VICTOR X4, multilabel plate reader) and O_2_ was expressed as the mean ± SD (%) of nanomoles per reduced cytochrome C per micrograms of protein compared to control [36].

### 2.11. NO Production

Nitric Oxide (NO) production was measured by Griess Assay (Promega Corporation, Madison, Wisconsin, United States). After 6 days of treatment, supernatants of basolateral BBB were mixed with equal volumes of Griess reagent and incubated in the dark at room temperature for 10 min; absorbance was measured by a spectrometer at 570 nm. NO production corresponded to the NO (μmol) produced after each stimulation by samples, each containing 1.5 μg of protein [37].

### 2.12. Mitochondrial Membrane Potential

The mitochondrial membrane potential was analyzed following manufacturer’s instructions of Oxygen Consumption/Mito membrane Potential Dual Assay Kit (Cayman Chemical Company, Ann Arbor, MI, United States) [38]. The mitochondrial membrane potential was measured using JC-1 aggregates at an excitation/emission of 560/590 nm and monomers at an excitation/emission of 485/535 nm in a fluorescence spectrometer (VICTOR X4, multilabel plate reader). The results are expressed as means ± SD (%) compared to control cells in both cell types.

### 2.13. ERK Activation Assay

ERK/MAPK activity was measured by the InstantOne™ ELISA (Thermo Fisher, Milan, Italy) on astrocytes lysates following the manufacturer’s instructions [36]. Briefly, 50 μL/well of astrocytes lysed with cell lysis buffer were tested in InstantOne ELISA microplate strips after 1 h at room temperature on a microplate shaker with the antibody cocktail. At the end, the detection reagent was added for 20 min and then stopped by adding stop solution. The strips were measured by a spectrometer (VICTOR X4 multilabel plate reader) at 450 nm. The results were expressed as mean absorbance (%) compared to control.

### 2.14. Western Blot

To perform Western blot analysis, 1 × 106 astrocytes plated in 6-well were lysed in ice with Ripa Buffer (50 mM Hepes, 150 mM NaCl, 0,1% SDS, 1% Triton X-100, 1% deoxycholate acid, 10% glycerol, 1.5 mM MgCl_2_, 1 mM EGTA, 1 mM NaF; Sigma-Aldrich, Milan, Italy) supplemented with 2 mM sodium orthovanadate and 1:100 mix Protease Inhibitor Cocktail (Sigma-Aldrich, Milan, Italy). 30 µg proteins were resolved on 8% and 15% SDS-PAGE gels. Brain tissue was also analyzed by Western blot to verify the mechanisms observed in cell culture. At the end of treatments brain tissue was excised out, washed in ice saline solution, weighed, cut in small pieces, and homogenized 100 mg tissue/300 µL with cold lysis buffer (0.1 M Tris, 0.01 M NaCl, 0.025 M EDTA, 1% NP40, 1% Triton X-100; Sigma-Aldrich, Milan, Italy) supplemented with 2 mM sodium orthovanadate, 0.1 M sodium fluoride (Sigma-Aldrich, Milan, Italy), 1:100 mix of protease inhibitors (Sigma-Aldrich, Milan, Italy), and 1:1000 phenylmethylsulfonyl fluoride (PMSF; Sigma-Aldrich, Milan, Italy), using an electric potter at 1,600 rpm for 2 min. The tissue extracts were centrifuged at 13,000 rpm for 20 min at 4 °C and 40µg of each lysate were resolved on 8% and 15% SDS- PAGE gel. Polyvinylidene difluoride (PVDF) membranes (GE Healthcare Europe GmbH, Milan, Italy) obtained from cell and brain tissue lysates were incubated overnight at 4 °C with specific primary antibody: anti-phospho-tyrosine receptor kinase B (p-TrkB, Tyr515; 1:250, Santa Cruz, CA, United States), anti-tyrosine receptor kinase B (trkB; 1:250, Santa Cruz, CA, United States), anti-Apolipoprotein E (apoE, E4; 1:250, Santa Cruz, CA, United States), anti-phospho-Sirtuin1 (pSIRT1, Ser47; 1:1000, Sigma-Aldrich, Milan, Italy), anti-Phospho-p44/p42 Mitogen-activated protein kinase (pERK/MAPK, Thr202/Tyr204; 1:1000, Euroclone, Milan, Italy), anti-p44/p42 Mitogen-activated protein kinase (ERK/MAPK; 1:1000, Euroclone, Milan, Italy) and anti-Phospho-Tau (pTau, Ser262; 1:250, Thermo Fisher Scientific, Waltham, MA, United States). In addition, in brain anti-BDNF (1:500, Sigma-Aldrich, Milan, Italy) and anti-β-Amyloid (APP, B-4, 1:500, Santa Cruz, CA, United States) were also investigated. All protein expressions were normalized to the specific total protein (if possible), verified through β-actin detection (1:5000, Sigma-Aldrich, Milan, Italy) and expressed as mean ± SD (%).

### 2.15. Animal Model

12-month-old mice wild type C57BL/6jOlaHsd of comparable age to an elderly human (about 80 years old) [39] purchased from Envigo++++ (Bresso, Italy), were used to confirm the effects of BDNF solutions in a complex model (*n* = 52). Starting from a new protocol to induce a spontaneous intake [40], we created a new rissole without bromophenol blue containing 1.2 pg/mL BDNF SKA or 25 ng/mL BDNF which is voluntary eaten by old mice. The quantity of rissoles was calculated considering the quantity of food and daily water normally taken by the animals [41]. The rissole preparation phases are summed up in Appendix A. The animals had access to food and water ad libitum and the experimental subjects were transferred to a single cage and kept in a single holding room and housed in a constant temperature of 21–22 °C, humidity of 5–55%, for 3 h [40,42]. Due to the short time taken to administer the rissole, mice showed no signs of social deprivation, such as increased aggressiveness. These signs have in fact been observed for periods of social deprivation of 6 h [43]. After this time, the rissole was added in the lower part of the cage, but the mouse had free access to food and water in the upper part. Time of stimulation started from the addition of the rissole. During the whole period of treatment, the mice were monitored to assess their health status. Serum was obtained from blood of intracardiac withdrawal after inducing anesthesia, and brain tissue was obtained after animal death. All experimental procedures on animals were reviewed and approved by the University Committee OPBA (Organismo preposto al benessere degli animali) in accordance with local ethical standards and protocols approved by national guidelines (Approval No. 41/2019-PR). Animals were randomized into four different times of treatment: untreated (12 animals, 4 for each times of treatment), 24 h (20 animals), 24 h plus 24 h (10 animals) and 6-day protocol (only one administration for 6 days, 10 animals). In particular, 24 animals were sacrificed after 24 h and 14 animals were sacrificed after 48 h (24 h with rissole plus 24 h without rissole administration but the animals had access to food and water ad libitum in the upper part of cage). Finally, to demonstrate the efficacy of treatment, 14 animals were sacrificed 6 days after the only administration of BDNF solution (6-day protocol). All groups were sacrificed at specific time points (24 h, 24 h plus 24 h and 6 days) by CO_2_ asphyxiation and blood drawn at the same time. The blood was centrifuged at 3500 rpm for 15 min at room temperature and the serum was conserved at −80 °C for subsequent experiments. In addition, the brain was removed, frozen and conserved at −80 °C for successive analysis by ELISA and Western blot on whole brain tissue lysates.

### 2.16. Statistical Analysis

Each part of the study is supported by at least 4 independent experiments both in vitro and in vivo. All results were analyzed by one-way analysis of variance (ANOVA) followed by Bonferroni post hoc test; data are expressed as mean ± SD of at least four independent experiments for each experimental protocol produced in triplicates. The percentage values were compared through Mann–Whitney U test. Comparisons between the two groups were performed using a two-tailed Student’s t-test. Multiple comparisons between groups were analyzed by two-way ANOVA followed by a two-sided Dunnett post hoc testing. *p*-Value < 0.05 was considered statistically significant.

## 3. Results

The effectiveness of BDNF SKA was investigated by both in vitro and in vivo experiments, comparing 1 pg/mL and 1.2 pg/mL to BDNF 50 ng/mL and 25 ng/mL, respectively.

### 3.1. The Potential Intestinal Absorption as Evaluated In Vitro

Since BDNF SKA can be used by oral administration in human, the in vitro intestinal absorption was investigated. An in vitro intestinal barrier model was carried out to understand the ability of 1 pg/mLe and comparable to that of 5 BDNF SKA compared to 50 ng/mL BDNF to cross the intestinal barrier and to become available to the body. Analyzing the volume in the basolateral compartment 1 pg/mL BDNF SKA showed a significant increase in absorption capacity compared to saline and comparable to that of 50 ng/ml of BDNF at each stimulation time (Table 1). In particular, the maximum Papp, the permeability constant value, was observed at 1 h of treatment with 1 pg/mL BDNF SKA (about 4.18 ± 0.1), supporting the hypothesis that BDNF SKA can cross the intestinal barrier.

Following a standard conversion to predict the human absorption after oral intake starting from the Papp values obtained from Caco-2 cells, the BDNF bioavailability in human (Table 1) showed an increase caused by 1 pg/mL BDNF SKA compared to saline solution and 50 ng/mL BDNF at each time of treatments and confirmed a higher level at 1 h of stimulation (about 4% compared to 50 ng/mL BDNF). These data confirm that 1 pg/mL BDNF SKA is able to cross the intestinal barrier and to has a good bioavailability compared to 50 ng/mL BDNF.

**Figure A2:**
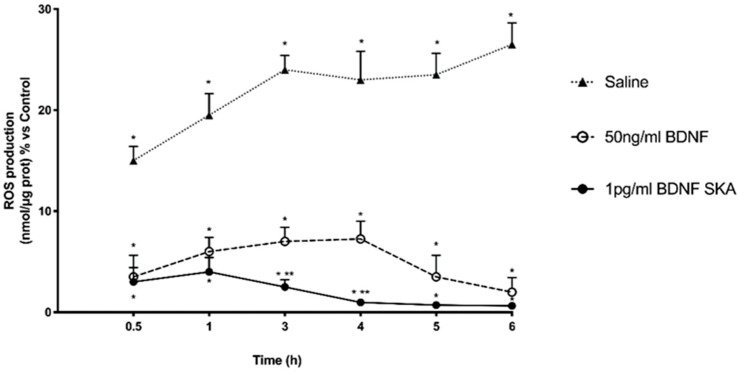
ROS production at intestinal barrier. Data are expressed as means ± SD (%) of five independent experiments performed in triplicates (0 line as control). Saline = saline solution. * *p* < 0.05 vs. control, ** *p* < 0.05 vs. 50 ng/mL BDNF.

Since safety is the main problem affecting human use, additional experiments on ROS production were performed on the intestinal barrier to exclude any adverse effects. Both 50 ng/mL BDNF and 1 pg/mL BDNF were able to maintain ROS at physiological levels (*p* > 0.05 vs. saline solution and control), supporting the safe use of this substance (Appendix B: Figure A2). Besides, the higher effect of 1 pg/mL BDNF compared to 50 ng/mL was observed after 3 and 4 h of treatment *(p* < 0.05; compared to saline solution, 50 ng/mL BDNF and control) demonstrating the best antioxidant action of BDNF SKA.

These data support the hypothesis that BDNF SKA is able to cross the intestinal barrier and reach the blood in the first 3–4 h after oral intake.

### 3.2. Permeability of BDNF SKA Through Blood–Brain Barrier (BBB)

**Figure 1:**
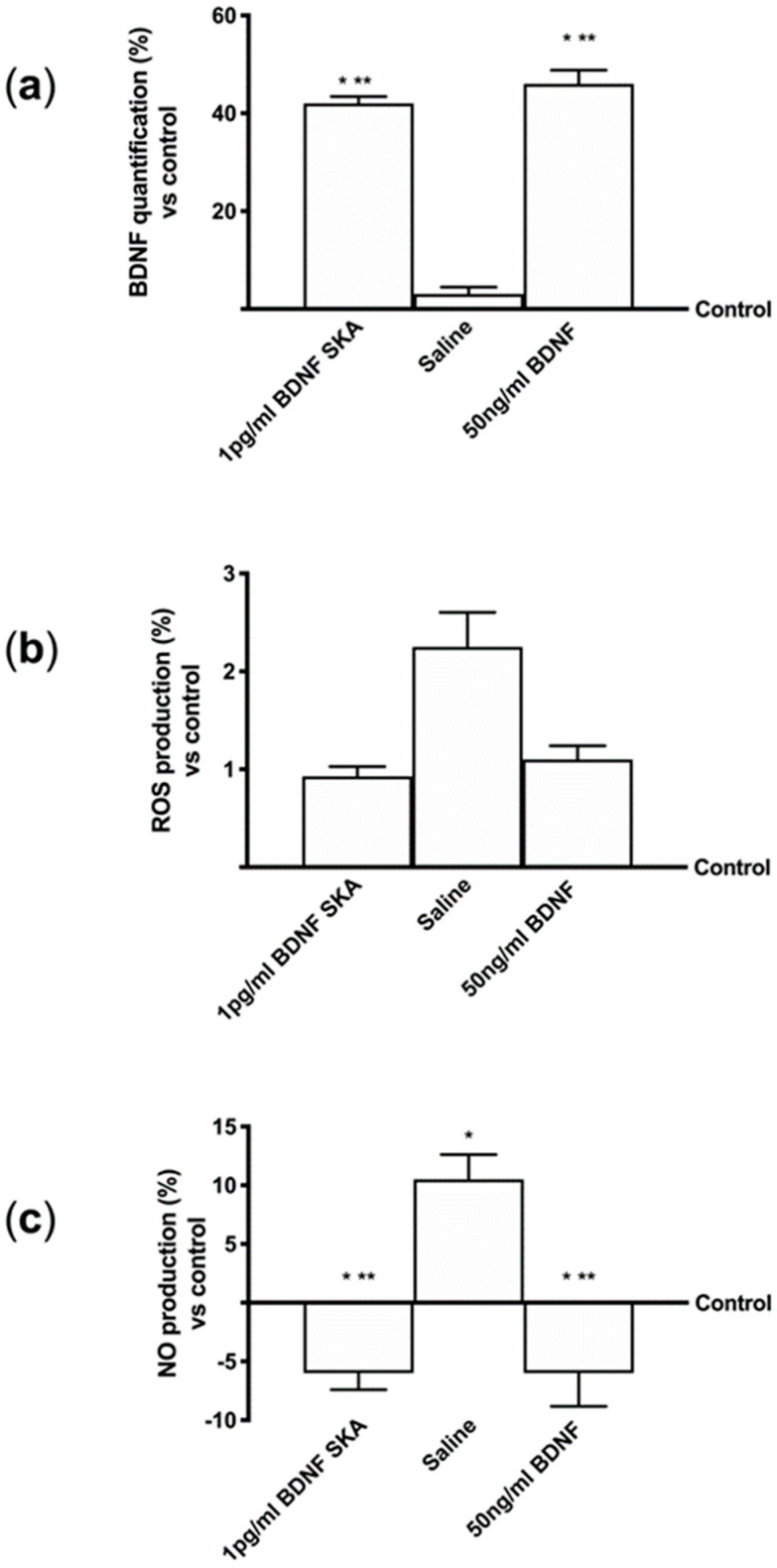
Analysis of the effects at blood–brain barrier (BBB) level. (**a**) Brain-derived neurotrophic factor (BDNF) quantification, (**b**) reactive oxygen species (ROS) production and (**c**) NO measurements are reported. Data are expressed as means ± SD (%) of four independent experiments performed in triplicates normalized to control (0 line as control). * *p* < 0.05 vs. control; ** *p* < 0.05 vs. saline solution.

Since the most important parameter after oral intake is the ability of BDNF to reach the brain tissue, more experiments were performed using the BBB in vitro model. The analysis on the basolateral volume of 1 pg/mL BDNF SKA and 50 ng/mL BDNF showed no significant difference between 1 pg/mL BDNF SKA and 50 ng/mL BDNF (Figure 1A), but the quantification showed a significant increase (*p* < 0.05) of both 1 pg/mL BDNF SKA and 50 ng/mL BDNF compared to control and to saline solution (about 41% and about 44%, respectively). These data suggest that only one treatment for six days appears to be more important to obtain a greater effect.

Furthermore, ROS production was analyzed in order to exclude any adverse effect caused by BDNF solutions. As shown in Figure 1B, there was no difference evident between 1 pg/mL BDNF SKA and 50 pg/mL BDNF and the effects of both solutions were not significant (*p* > 0.05) compared to control, indicating a physiological ROS production. These data confirmed the hypothesis of the higher effectiveness of administration protocol.

Since maintaining the balance of oxidative condition is an important parameter to preserve the integrity of brain cells, some additional experiments were carried out to analyze NO production within the BBB (Figure 1C). At basolateral level, the NO production induced by protocol A was significantly reduced compared to control (*p* < 0.05). In particular, there was no significant difference between 1 pg/mL BDNF SKA and 50 ng/mL BDNF treatment, suggesting that BDNF solutions were not cytotoxic.

These results suggest that 1 pg/mL BDNF SKA had a similar effect to 50 ng/mL SKA despite the different concentrations and that only one treatment is able to induce a beneficial effect. Finally, BDNF SKA is confirmed to act without any adverse effect at the neuronal level.

### 3.3. Topic Action of BDNF SKA on Monolayer Culture

**Figure 2:**
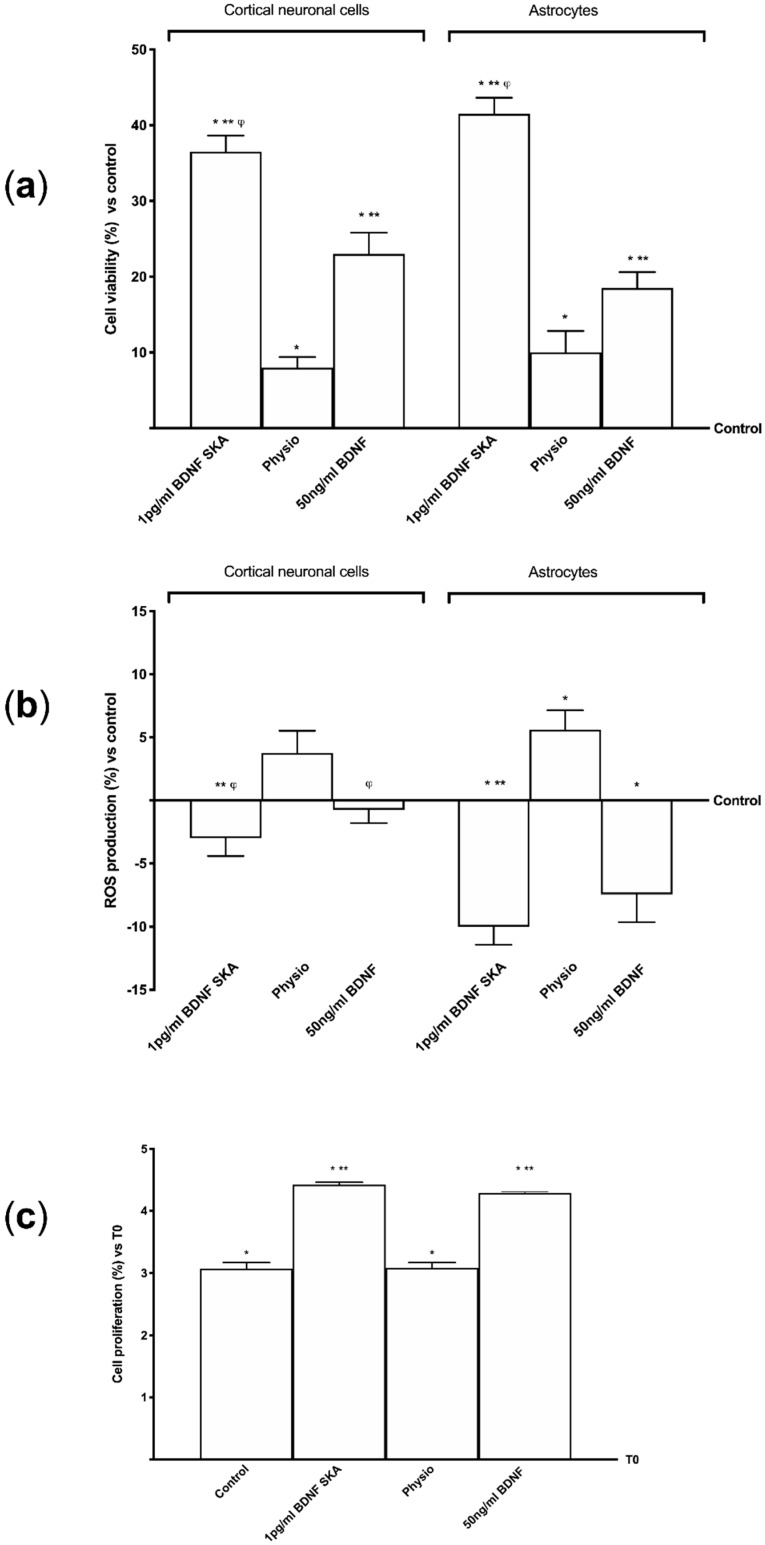
Effects of BDNF solutions on primary cortical neuronal cells and astrocytes. (**a**) Cell viability and (**b**) ROS production measured on both cell types. (**c**) The effects on astrocytes proliferation are shown. Data are expressed as means ± SD (%) of four independent experiments performed in triplicates normalized to control (0 line as control). * *p* < 0.05 vs. control; ** *p* < 0.05 vs. saline solution; ^φ^ *p* < 0.05 vs. the same treatments between primary cortical neuronal cells and astrocytes in the same protocol.

BDNF has been demonstrated to be able to act on both cortical neuronal cells and astrocytes, additional experiments were performed in order to investigate cell viability and ROS production in these monolayer cultured cells. In particular, 1 pg/mL of BDNF SKA induced a significantly greater cell viability (Figure 2A) both in primary cortical neuronal cells and in astrocytes compared to control (*p* < 0.05) and 50 ng/mL of BDNF (in neuronal cells approximately 124% and 75%, respectively, in the astrocytes approximately 58.6% and 171%, respectively). These findings suggest that though BDNF SKA was used at lesser concentrations it was able to determine a greater effect on cell viability compared to the higher concentration; furthermore these results support the hypothesis of the effectiveness of the single administration compared to multiple administrations.

Since an important contributing factor to brain aging is the exaggerated ROS production, additional experiments on ROS production were performed on both cell types following both protocols of treatments (Figure 2B). Results obtained from these experiments show that 1 pg/mL BDNF SKA was able to maintain ROS production within physiological range in both cortical neuronal cells and astrocytes (*p* > 0.05 vs. control). These data suggest that BDNF SKA is able to maintain the redox balance even in monolayer cultured cells. Although 50 ng/mL BDNF also shows similar properties, the effect was significantly lower compared to 1 pg/mL BDNF SKA.

Basing on previous observation of viability and ROS production in astrocytes, the involvement of BDNF solutions in cell proliferation was also investigated by crystal violet staining. As reported in Figure 2C, 1 pg/mL BDNF SKA treatment was able to increase the proliferation of astrocytes (*p* < 0.05) compared to control (about 43.9% and 13.4%, respectively) and to saline solution (about 43.5% and 29.6%, respectively). In addition, the importance of the concentration used was confirmed as well. Indeed, 1 pg/mL BDNF SKA is more effective than 50 ng/mL BDNF (about 3.1%).

### 3.4. Intracellular Pathways Activated by BDNF SKA on Monolayer Culture

Since all results reported above show a better influence in astrocytes compared to the same protocol on neurons, the intracellular pathways involved were investigated only on astrocytes.

In this phase of the study, the intracellular pathways involved in the previously observed effects were studied. The effects induced by 1 pg/mL BDNF SKA on ApoE expression, SIRT1 phosphorylation, ERK/MAPK pathway and the levels of activation of the BDNF receptor, TrkB, were studied.

**Figure 3:**
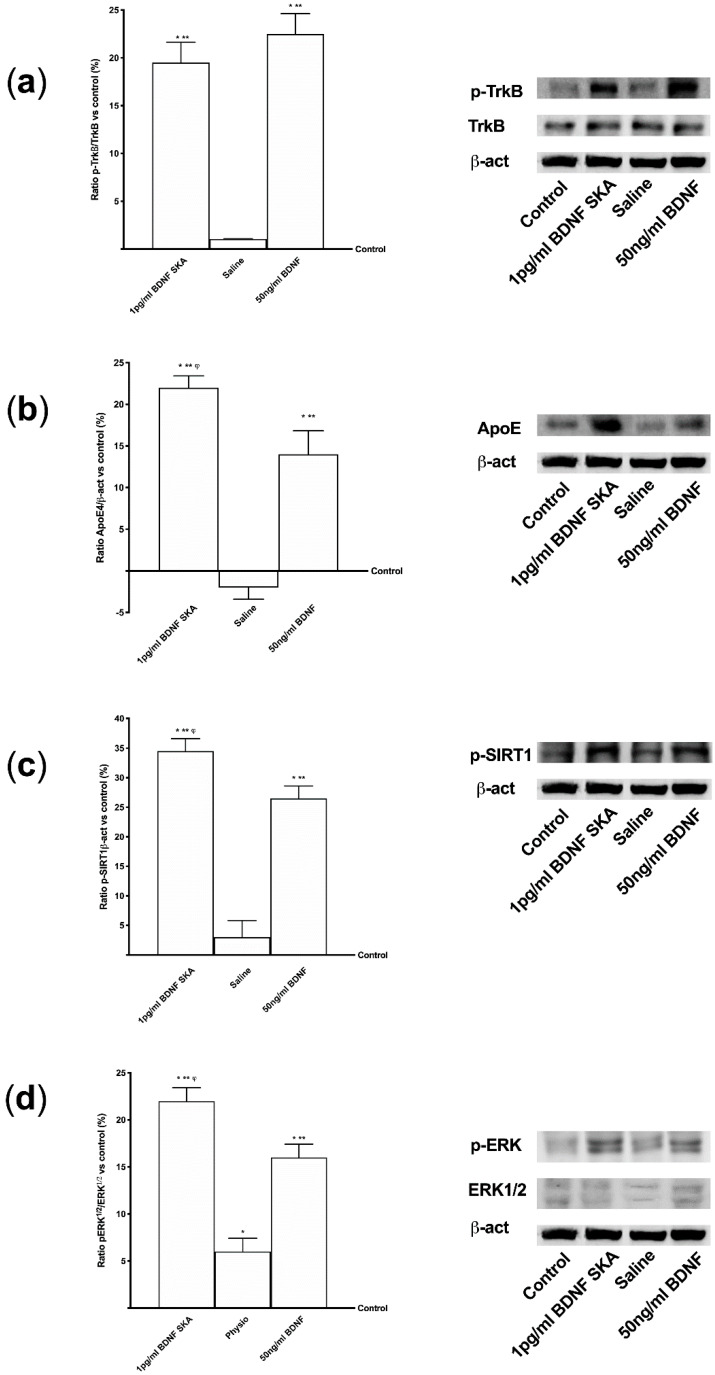
Analysis of intracellular pathways activated by BDNF solutions in astrocytes. In the left column densitometric analysis and in the right the examples of Western blot are reported. (**a**) TrkB receptor, (**b**) ApoE(4), (**c**) SIRT1 and (**d**) ERK/MAPK expressions are shown. Data are expressed as means ± SD (%) of five independent experiments normalized on specific total protein if possible and verified by β-actin detection. * *p* < 0.05 vs. control; ** *p* < 0.05 vs. saline solution; ^φ^ *p* < 0.05 vs. 50 ng/mL BDNF.

As reported in Figure 3A, BDNF solutions seemed to act by the TrkB receptor. No significant differences were observed between 50 ng/mL BDNF and 1pg/mL BDNF SKA, indicating the ability of 1 pg/mL BDNF SKA to lead TrkB receptor to exert its effects despite the low dose used. As far as the ApoE expression is concerned, was significantly increased by 1 pg/mL BDNF SKA (*p* < 0.05) compared to 50 ng/mL BDNF (about 80%) (Figure 3B). As illustrated in Figure 3C, phosphorylation of SIRT1 induced by 1 pg/mL BDNF SKA (*p* < 0.05) was increased compared to 50ng/mL BDNF (about 30%), supporting the efficacy of the dosage to increase the presence of this molecule.

Finally, as shown in Figure 3D, BDNF has been observed to increase the ERK1/2 expression and the greater effect was obtained with 1 pg/mL BDNF SKA (*p* < 0.05) compared to 50 ng/mL BDNF (about 37%).

**Figure 4:**
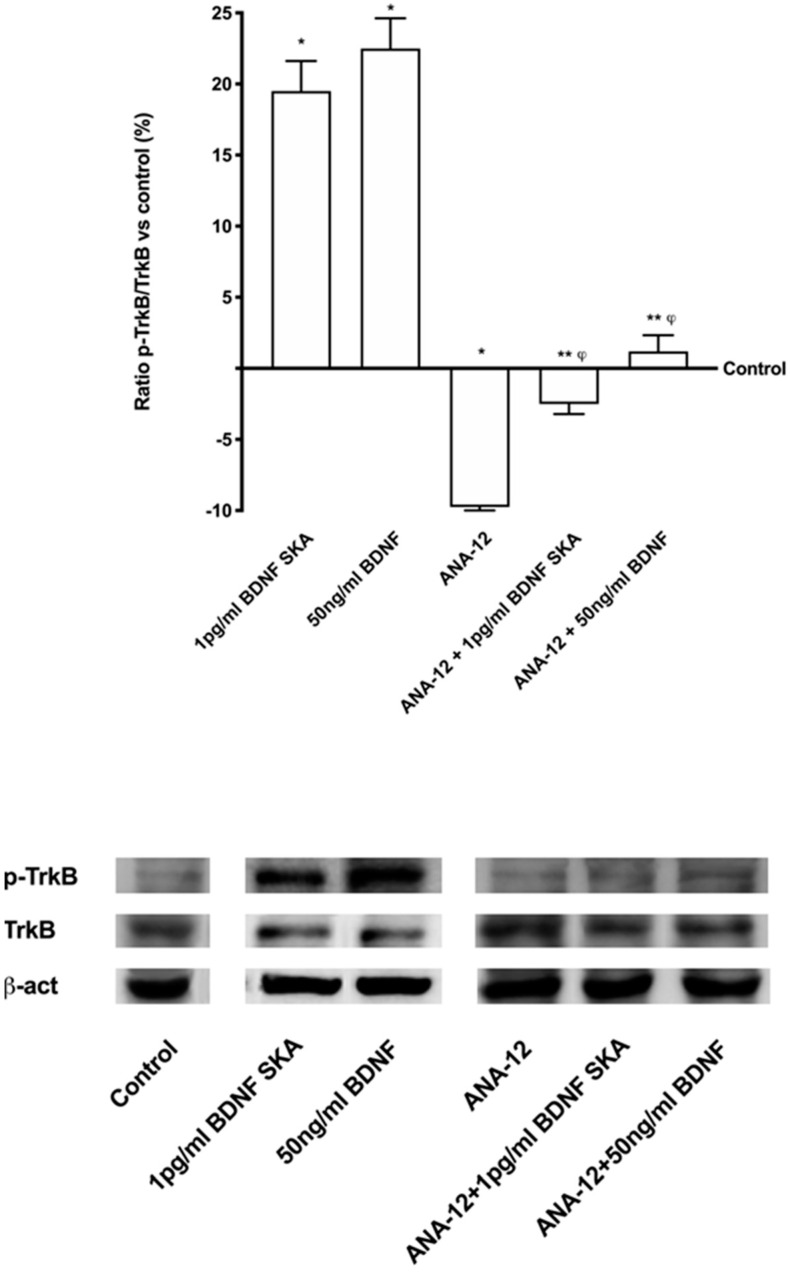
Analysis of TrkB receptor under blocking condition on astrocytes. In the upper panel densitometric analysis and in the lower panel an example of Western blot is reported. Data are expressed as means ± SD (%) of five independent experiments normalized on specific total protein and verified by β-actin detection. ANA-12 = 1 µg/mL ANA-12. * *p* < 0.05 vs. control; ** *p* < 0.05 ANA-12; ^φ^ *p* < 0.05 vs. the same treatments without ANA-12.

Additional experiments were performed to confirm the involvement of the TrkB receptor in previously observed effects, using a pre-treatment with the selective TrkB antagonist ANA-12 (1 µg/mL) on astrocytes. As reported in Figure 4, in the presence of both BDNF solutions, TrkB expression was abolished by the pre-treatment with 1 µg/mL ANA-12, confirming that both BDNF solutions acted through the TrkB receptor to explain their effects on astrocytes. These data confirm the importance of the dosage and the protocol of treatment to obtain a beneficial effect on astrocytes under physiological conditions.

### 3.5. Effects of BDNF Solutions Under Oxidative Conditions

**Figure 5:**
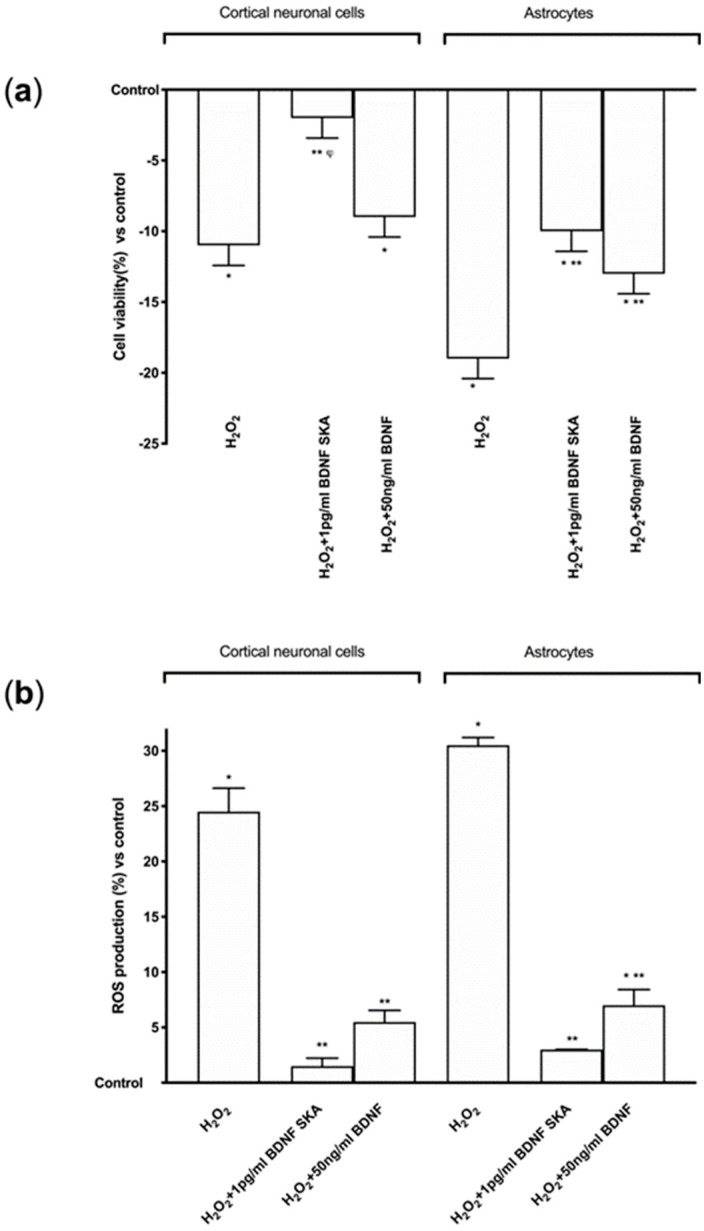
Cell viability and ROS production on both protocols in primary cortical neuronal cells and astrocytes under oxidative condition. (**a**) Cell viability and (**b**) ROS production measured in primary cortical neuronal cells (on the left) and astrocytes (on the right) after treatments. H_2_O_2_ = 200µM H_2_O_2_ added 30 min before stimulations. Data are expressed as means ± SD (%) of five independent experiments performed in triplicates normalized to control (0 line as control). * *p* < 0.05 vs. control; ** *p* < 0.05 vs. H_2_O_2_; ^φ^ *p* < 0.05 vs. H_2_O_2_+50 ng/mL BDNF in the same cells.

Cell viability and ROS production were evaluated in cortical neuronal cells and astrocytes in order to understand the potential aging-prevention mechanism of 1 pg/mL BDNF SKA and 50 ng/mL BDNF 50 ng/mL under oxidative conditions. Exposure to 200 μM H_2_O_2_, in both cell types significantly reduced (*p* < 0.05) cell viability compared to control (Figure 5A), indicating cell loss caused by oxidative injury. Conversely, following post-treatment with 1 pg/mL BDNF SKA and 50 ng/mL BDNF, cell viability increased in a different manner between cell types. Indeed, only in astrocytes did both BDNF solutions significantly increase cell viability (*p* < 0.05), but the main effect was observed with 1 pg/mL BDNF SKA in both cell types (*p* < 0.05 vs. H_2_O_2_ alone), confirming the importance of doses and posology also under pathological conditions.

Additional experiments on ROS production were performed. Exposure of cortical neuronal cells and astrocytes to 200 μM H_2_O_2_ significantly increased the intracellular ROS production compared to control (*p <* 0.05), as illustrated in Figure 5B, confirming the presence of oxidative damage. Post-treatment with 1 pg/mL BDNF SKA and 50 ng/mL BDNF in both cell types caused a significant reduction of ROS production (*p <* 0.05) compared to H_2_O_2_ alone, supporting the hypothesis of the importance of BDNF during degeneration to prevent cell loss. These data suggest that 1 pg/mL BDNF SKA is able to counteract the damage induced by oxidative stress with greater effectiveness compared to 50 ng/mL BDNF.

**Figure 6:**
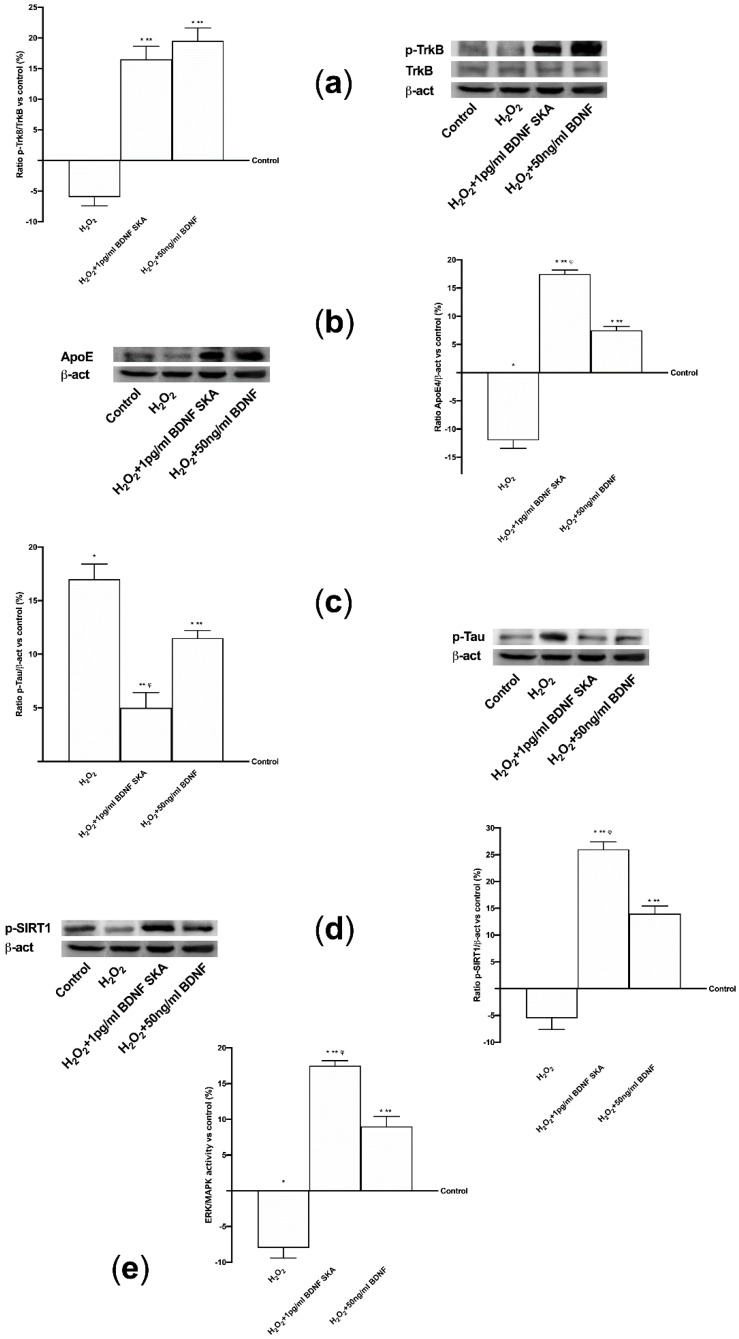
Analysis of intracellular pathways activated by BDNF solutions in astrocytes under oxidative condition. Kinase activity, densitometric analysis and Western blot are reported. (**a**) TrkB receptor, (**b**) ApoE(4), (**c**) Tau, and (**d**) SIRT1 expressions and (**e**) ERK/MAPK activity. Data are expressed as means ± SD (%) of five independent experiments and the densitometric analyses are normalized on specific total protein if possible and verified by β-actin detection. * *p* < 0.05 vs. control; ** *p* < 0.05 vs. H_2_O_2_; ^φ^ *p* < 0.05 vs. H_2_O_2_+50 ng/mL BDNF.

Since the data obtained from the two cell types were comparable, the analysis of intracellular pathways under oxidative conditions was conducted only on astrocytes. BDNF solutions exerted their biological actions by improving TrkB receptor expression even in the presence of H_2_O_2_, as shown in Figure 6A, (*p* < 0.05 versus control). No significant changes were observed between the two BDNF solutions. Moreover, the expressions of ApoE and of Tau, an important protein that modulates the stability of axonal microtubules, were analyzed. As reported in Figure 6B, the stimulation with H_2_O_2_ alone caused a significant decrease of ApoE expression compared to control (*p <* 0.05), indicating a loss of neuroplasticity. Conversely, the treatments with 1pg/mL BDNF SKA and 50 ng/mL BDNF repaired the damage and the expression of ApoE was increased. In particular the main effect was observed by 1 pg/mL BDNF SKA compared to 50 ng/mL BDNF (*p <* 0.05, about ten fold larger). The presence of the damage was confirmed by Tau phosphorylation, as reported in Figure 6C, where the level was even higher than control (*p <* 0.05). After treatment with 1 pg/mL BDNF SKA compared to 50 ng/mL BDNF the phosphorylation was significantly reduced (*p <* 0.05, about 56%), indicating the efficacy of 1 pg/mL BDNF SKA to restore damage. In addition, the analysis of SIRT1 confirms the protection exerted by 1 pg/mL BDNF SKA and 50 ng/mL BDNF against H_2_O_2_ damage (Figure 6D); 1 pg/mL BDNF SKA and 50 ng/mL BDNF were able to induce a significant increase in SIRT1 phosphorylation compared to H_2_O_2_ alone (*p <* 0.05) and to control (*p <* 0.05). However, the main effect was shown by 1 pg/mL BDNF SKA compared to 50ng/mL BDNF (about 85%). All these findings support the hypothesis that treatment with BDNF SKA can protect neuronal cells from the damage induced by the aging process better than high dose BDNF. Finally, as reported in Figure 6E, the activation of TrkB induced cells’ survival by the involvement of ERKs/MAPK; indeed, 1 pg/mL BDNF SKA and 50 ng/mL BDNF added after the injury were able to induce a significant increase on ERK activity compared to H2O2 alone (*p <* 0.05) and to control (*p <* 0.05). However, the main effect was observed in presence of 1 pg/mL BDNF SKA compared to 50 ng/mL BDNF (about 90%).

### 3.6. Daily Duration of the Effects of BDNF Solutions on Neurons and Astrocytes

Since BDNF can be used as a dietary supplement in humans, some experiments were carried out to better clarify the optimal dosing schedule. The effects of 1 pg/mL BDNF SKA and 50 ng/mL BDNF during 24 h on both cell types were studied analyzing BDNF concentration, cell viability and mitochondrial potential.

**Figure 7:**
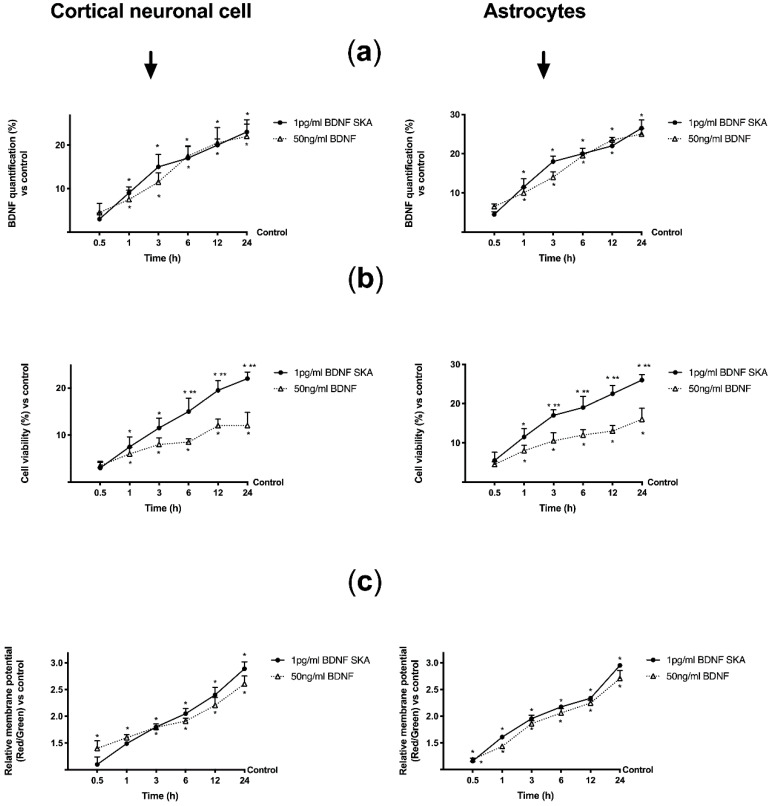
Effects of BDNF solutions within 24h on primary cortical neuronal cells and astrocytes. In the left column primary cortical neuronal cells and in the right column astrocytes are shown. (**a**) BDNF quantification and (**b**) cell viability measured after BDNF treatments. Data are expressed as means ± SD (%) of five independent experiments performed in triplicates normalized to control (0 line as control). (**c**) The mitochondrial membrane potential is investigated in the same condition. Data are expressed as means ± SD of five independent experiments performed in triplicates normalized to control (1 line as control). * *p* < 0.05 vs. control; ** *p* < 0.05 between 1 pg/mL BDNF SKA and 50 ng/mL BDNF.

As reported in Figure 7A, treatments with 1 pg/mL BDNF SKA and 50 ng/mL BDNF on both cell types caused a similar time-dependent increase in BDNF concentration. This effect showed significance from 1 h, compared to control (*p* < 0.05), and the maximum effect was observed at 24 h (23% and 22% compared to control, respectively, in neurons; about 26% and 25% compared to control, respectively, in astrocytes). No significant changes between the two BDNF solutions were observed, indicating the comparable effectiveness of low dose SKA to high-concentration BDNF.

However, 1 pg/mL BDNF had better tolerability, demonstrated by better cell viability (*p* < 0.05) compared to 50 ng/mL BDNF. This effect was significant starting from 6 h in neurons and 3 h in astrocytes, with a maximum effect on both types of cells at 24 h (about 80% and about 60% vs. 50 ng/mL, respectively), as shown in Figure 7B.

Since cell viability depends on mitochondrial activity, the analysis of mitochondrial potential variation was performed (Figure 7C). Both BDNF solutions modulated mitochondrial potential in a time-dependent manner with a significant increase from 3 h in neurons and from 30 min in astrocytes compared to control (*p* < 0.05). However, no significant changes were observed between the two BDNF solutions.

These results therefore allow us to state that low dose BDNF SKA administration has more beneficial effects on neurons and astrocytes than high dose BDNF, prolonged over time to cover 24 h.

### 3.7. Analysis of Bioavailability of BDNF Solutions and Their Effects in Mouse Brain

**Figure 8:**
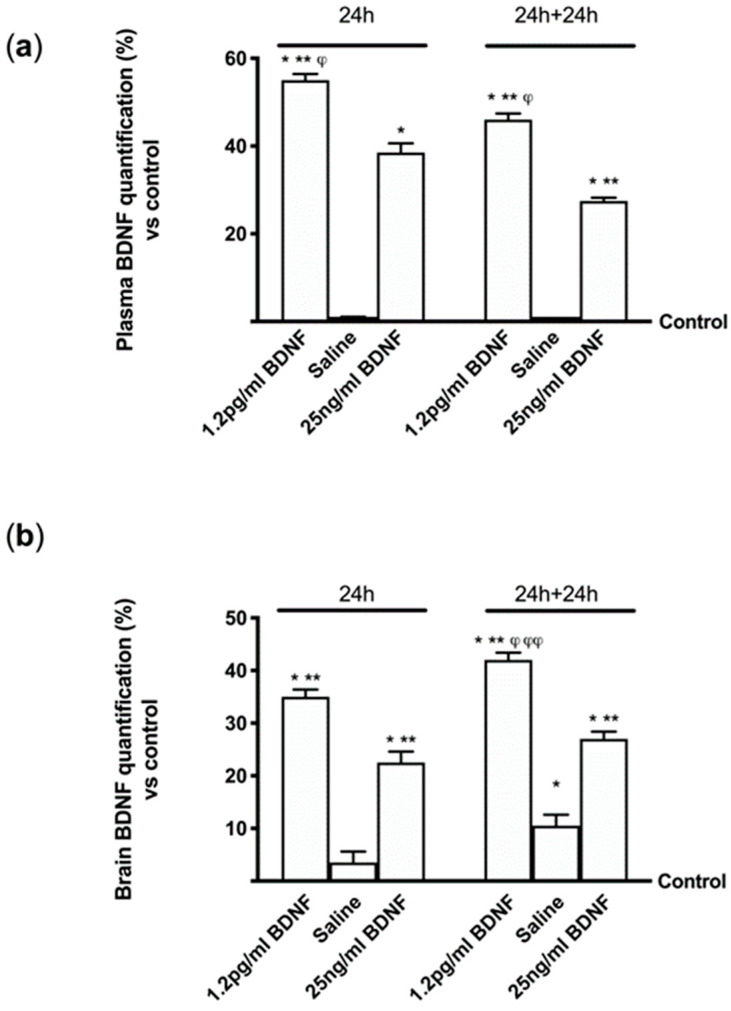
BDNF quantification in mice in serum and brain tissue. (**a**) Serum quantification and (**b**) BDNF quantification in brain tissue are reported. Each graph contains on the left the results obtained at 24h (12 animals) and on the right (12 animals) at 24 h plus 24 h (24 h+24 h). Data are expressed as means ± SD (%) normalized to control (0 line as control). * *p* < 0.05 vs. control; ** *p* < 0.05 vs. saline solution; ^φ^ *p* < 0.05 vs. the same treatments in the same time of administration; ^φφ^ *p* < 0.05 vs. the same treatments at 24 h and 24 h plus 24 h.

To confirm the ability of BDNF solutions to cross enterohepatic circle and the blood–brain barrier and act in brain tissue, some experiments were performed in vivo using wild type C57BL mice. Since in humans the administration would be daily, in some experiments 1.2 pg/mL BDNF SKA and 25 ng/mL BDNF were administered to animals by rissoles and BDNF concentration in both serum and brain tissues were analyzed after 24 h. In addition, to verify the stability of the effects, additional experiments were carried out adding 24h without stimulations. As reported in Figure 8A, 1.2 pg/mL BDNF SKA had a greater ability to get through the enterohepatic circle compared to 25 ng/mL BDNF (about 43%) and to control (*p* < 0.05) at 24 h. Moreover, 1.2 pg/mL BDNF SKA tended to remain in blood circulation longer (at least 24 h longer), compared to 25 ng/mL BDNF (about 68%). Since BDNF is present in blood, it is important to verify its presence also in brain tissue (Figure 8B). In administration of both 1.2 pg/mL BDNF SKA and 25 ng/mL BDNF, it was able to enter the brain, as illustrated by BDNF quantification analysis (*p* < 0.05 vs. control). In addition, 1.2 pg/mL BDNF SKA was able to remain for a longer time (24 h plus 24 h) in brain tissue compared to 25 ng/mL BDNF (about 55%, *p* < 0.05) and 1.2 pg/mL BDNF SKA at 24 h (about 20%, *p* < 0.05). These findings demonstrate the importance of doses and posology of administration of BDNF SKA to induce a better influence on brain tissue.

**Figure 9:**
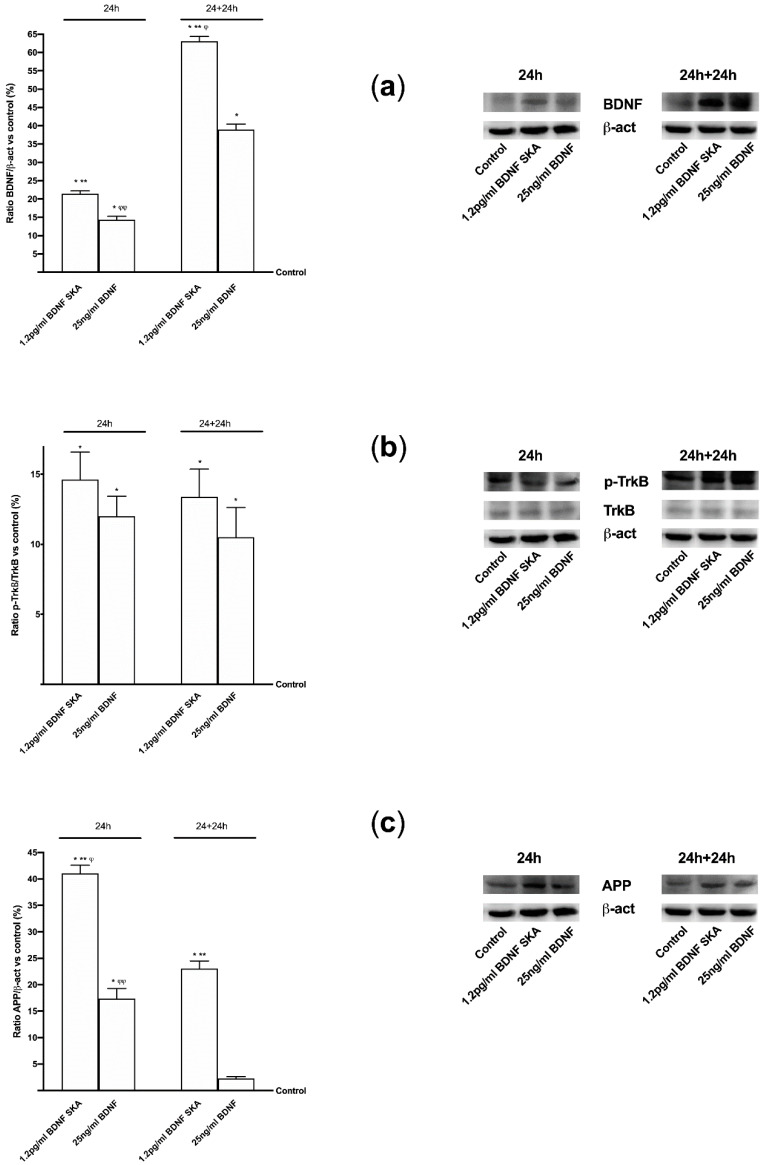
Western blot and densitometric analysis of BDNF protein (**a**), TrkB (**b**) receptor and (**c**) APP protein expressions in brain tissue. In the left column densitometric analysis and in the right the examples of Western blot are reported. Each graph contains on the left the results obtained at 24 h (12 animals) and on the right (12 animals) at 24 h plus 24 h (24 h+24 h). Data are expressed as means ± SD (%) of independent experiments normalized on specific total protein if possible and verified by β-actin detection. * *p* < 0.05 vs. control; ** *p* < 0.05 vs. 25 ng/mL BDNF at the same time of administration; ^φ^ *p* < 0.05 vs. 1.2 pg/mL BDNF SKA between 24 h and 24 h plus 24 h; ^φφ^ *p* < 0.05 vs. 25 ng/mL BDNF between 24 h and 24 h plus 24 h.

To verify whether the mechanism activated by BDNF solutions is the same as the one observed in cells during in vitro experiments, the effects of 1.2 pg/mL BDNF SKA and 25 ng/mL BDNF on some main markers were investigated by Western blot. Since BDNF is necessary for survival of neurons in the brain, after encoding by this gene its expression was investigated, as reported in Figure 9A. 1.2 pg/mL BDNF SKA and 25 ng/mL BDNF both at 24 h and 24 h plus 24 h were able to induce the expression of BDNF compared to control (*p* < 0.05), indicating a better influence of stimulations. Moreover, 1.2 pg/mL BDNF SKA at 24 h and 24 h plus 24 h caused a significant increase compared to and 25 ng/mL BDNF (about 50% and about 62%, respectively), indicating the induction of endogenous production of BDNF by physiological mechanism, as shown by the significant increase induced by 1.2 pg/mL BDNF SKA at 24 h plus 24 h with respect to at 24 h (*p* < 0.05, about 24%).

These effects were mediated by the TrkB receptor, which was expressed in a similar manner in both times of treatment between 1.2 pg/mL BDNF SKA and 25 ng/mL BDNF (*p* < 0.05 vs. control, Figure 9B). Since β-Amyloid precursor protein (APP) plays a central role, the beneficial effects exerted by both BDNF solutions were also assessed by the quantification of APP, as shown in Figure 9C. 1.2 pg/mL BDNF SKA and 25 ng/mL BDNF increased APP compared to control (*p* < 0.05) at 24h and 1.2 pg/mL BDNF SKA seemed to have a greater effect compared to 25 ng/mL BDNF (about 2.5 times). In addition, the APP activity at 24 h plus 24 h demonstrated the physiological action of 1.2 pg/mL BDNF SKA compared to 25 ng/mL BDNF (*p* < 0.05), indicating a better regulation exerted by 1.2 pg/mL BDNF SKA on central nervous tissue.

**Figure 10:**
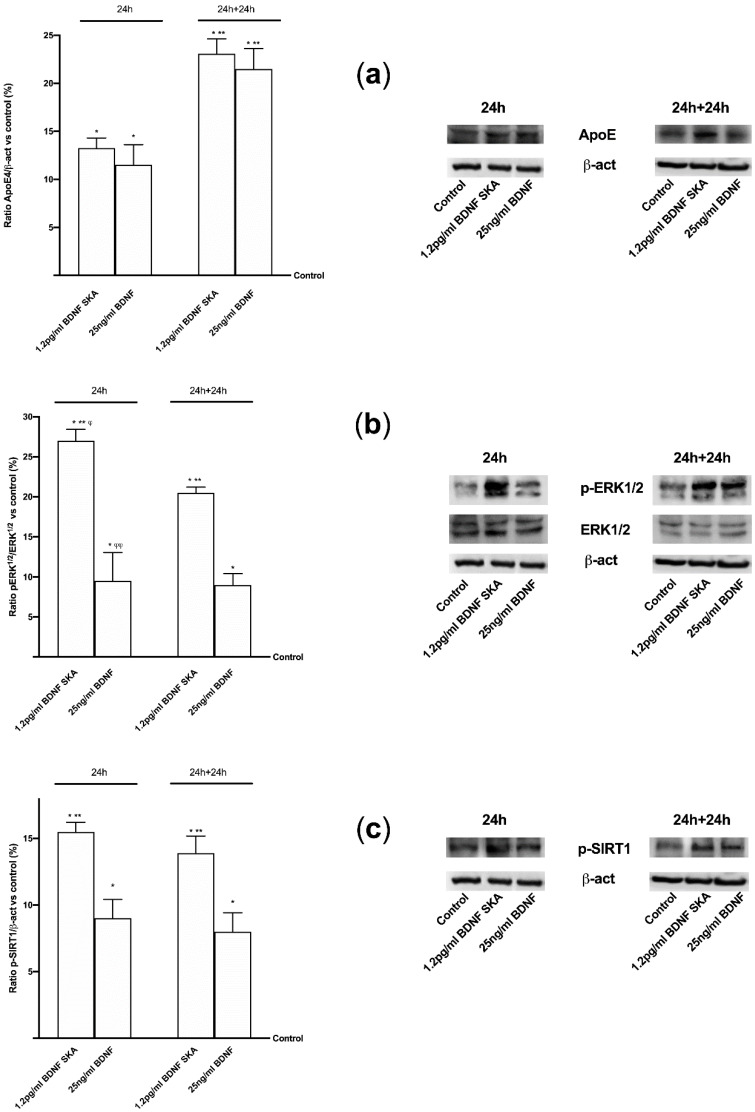
Western blot and densitometric analysis of ApoE (**a**), ERK/MAPK (**b**,**c**) SIRT1 expressions in brain tissue. In the left column densitometric analysis and in the right the examples of Western blot are reported. Each graph contains on the left the results obtained at 24 h (12 animals) and on the right (12 animals) at 24 h plus 24 h (24 h + 24 h). Data are expressed as means ± SD (%) of independent experiments normalized on specific total protein if possible and verified by β-actin detection. * *p* < 0.05 vs. control; ** *p* < 0.05 vs. 25 ng/mL BDNF at the same time of administration; ^φ^ *p* < 0.05 vs. 1.2 pg/mL BDNF SKA between 24 h and 24 h plus 24 h; ^φφ^ *p* < 0.05 vs. 25 ng/mL BDNF between 24 h and 24 h plus 24 h.

Moreover, since in vivo and in vitro studies suggested that ApoE may drive neurodegeneration through an Aβ-dependent mechanism, ApoE expression was assessed as well. As reported in Figure 10A, a significant increase of ApoE expression compared to control (*p* < 0.05) was observed in the presence of both 1.2 pg/mL BDNF SKA and 25 ng/mL BDNF in both time points, indicating a positive effect of BDNF on central nervous tissue. Moreover, the activation of TrkB by 1.2 pg/mL BDNF SKA and 25 ng/mL BDNF was able to induce a significant increase in ERKs expression compared to control (*p* < 0.05) in both time points. Therefore, a potential role of BDNF in tissue recovery through the involvement of ERKs/MAPK (Figure 10B) can be hypothesized. These findings support what was observed in astrocytes. However, the main effect was observed at 24 h in the presence of 1.2 pg/mL BDNF SKA compared to 25 ng/mL BDNF (*p* < 0.05, about three fold) and to 1.2 pg/mL BDNF SKA at 24 h plus 24 h (*p* < 0.05, about 30%). The last test of this series of experiments concerned the study of the expression of SIRT1. The analysis of SIRT1 confirms the beneficial effects exerted by 1.2 pg/mL BDNF SKA and 25 ng/mL BDNF (Figure 10C). In both 24 h and 24 plus 24 h, 1.2 pg/mL BDNF SKA and 25 ng/mL BDNF were able to induce a significant increase on SIRT1 phosphorylation compared to control (*p* < 0.05). However, the main effect was shown by 1.2 pg/mL BDNF SKA on both time points compared to 25 ng/mL BDNF (about 70% and 73%, respectively, *p* < 0.05). All these findings support the hypothesis that treatment with BDNF SKA can induce physiological mechanisms potentially able to slow down degeneration and protect brain during time.

### 3.8. Effects of BDNF Solutions in Mouse Brain During Time

**Figure 11:**
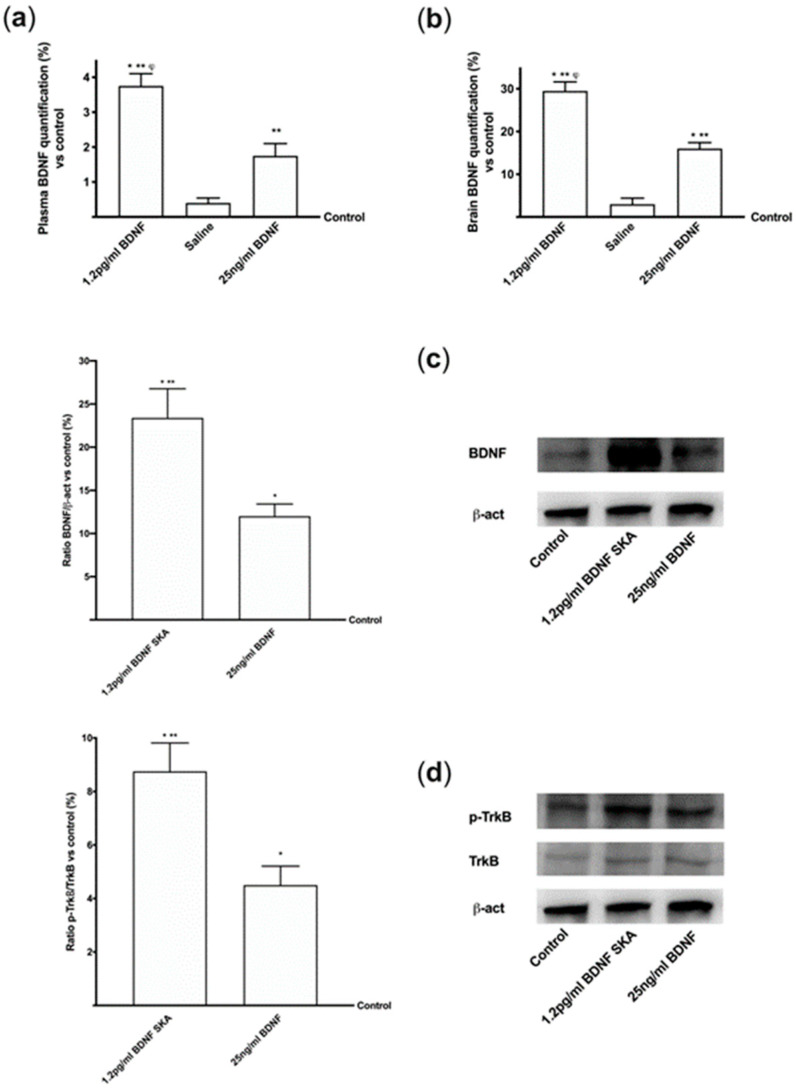
BDNF quantification and intracellular pathways in brain measured in the 6-dayprotocol. (**a**) Serum and (**b**) brain tissue BDNF quantification in mice. Data are expressed as means ± SD (%) of *n* = 7 independent experiments normalized to control value (0 line). * *p* < 0.05 vs. control; ** *p* < 0.05 vs. saline solution; ^φ^ *p* < 0.05 vs. 25ng/mL BDNF. (**c**) BDNF and (**d**) TrkB receptor expressions in mice brain reported as densitometric analysis (on the left) and examples of Western blot (on the right). Data are expressed as means ± SD (%) of *n* = 7 independent experiments normalized on specific total protein if possible and verified by β-actin detection. * *p* < 0.05 vs. control; ** *p* < 0.05 vs. 25 ng/mL BDNF.

To verify whether the efficacy of BDNF SKA was maintained for a long time, the mice were treated following the 6-day protocol previously used in cells experiments. As reported in Figure 11A, the administration of 1.2 pg/mL BDNF SKA and 25 ng/mL BDNF maintained the serum BDNF levels up to six days compared to control (*p* < 0.05). Besides, 1.2 pg/mL BDNF SKA was able to maintain high BDNF level compared to 25 ng/mL BDNF (about two fold higher). Similarly, the administration of 1.2 pg/mL BDNF SKA demonstrated better effectiveness (*p* < 0.05) compared to 25 ng/mL BDNF (about 80%) at brain tissue level (Figure 11B). These data suggest that BDNF SKA tends to remain present for a long time in brain tissue even in the absence of treatment, by triggering its physiological production better than BDNF at a high dose.

To confirm this, some additional experiments were performed to analyze BDNF protein (Figure 11C) and TrkB receptor by Western blot (Figure 11D). Both proteins show a significant increase at six days after both BDNF solutions, but 1.2 pg/mL BDNF SKA exerted a significant increase compared to 25 ng/mL BDNF (about two fold higher for each one, respectively, *p* < 0.05). These findings support the hypothesis of a fine endogenous regulation exerted by BDNF SKA on brain maintenance and function.

## 4. Discussion

Today neurodegenerative disorders are considered chronic and incurable conditions, whose disabling effects can last for years or decades. This represents a huge burden of suffering for patients and costs for health organizations. Optimal cognitive functions are linked to an efficient neuronal plasticity and the ability of neurons or glial cells to improve the efficacy of the synapses through biochemical and morphological changes, both at a dendritic and axonal level [44]. However, as reported by many studies, this ability shows a marked age-related decrease [15]. At present, treatments available for these diseases are mostly symptomatic or palliative and include neurotransmitter modulators, hormonal therapies, anti-inflammatory drugs, deep brain stimulation and herbal products. Therefore, there is an urgent need to develop new solutions able to restore the physiological functions of the brain tissue. Moreover, one of the main problems concerning the administration of active ingredients into the central nervous system is the crossing of the blood–brain barrier. Modern drug delivery systems can consist both of biodegradable and non-biodegradable formulations, which offer advantages in terms of protection, absorption, penetration and distribution of active ingredients. For this reason, the use of molecules already known for their exclusive functions within the brain and consequently physiologically predisposed to easily cross blood–brain barrier can be considered a valid option.

Recently, researchers’ attention has focused on the involvement of the neurotrophic factors in the development of neuronal decay. Currently, it is common knowledge that there are three main neurotrophic factors: the brain-derived neurotrophic factor (BDNF), the nerve growth factor and the glial cell-derived neurotrophic factor [45]. BDNF in particular is associated with the modulation of neuroplasticity, which promotes the health of nervous tissue and also has the ability to counteract the effects of pro-inflammatory cytokines, which are key factors in neurodegenerative processes [46].

In recent years, the possibility of using a growth factor therapy has been hypothesized by exploiting the growing information that research has accumulated, mainly on BDNF. Indeed, BDNF seems to have a real therapeutic potential, based on the observation that in many disorders of the nervous system serum BDNF levels are altered [47]. However, a major problem is the delivery of the molecule to the affected cells. Although numerous studies have explored the possibility of administering BDNF through several approaches, such as gene therapy vectors, the development of mimetic peptides or even through direct administration into the nervous system, the results are still penalized by the lack of ease of use [47]. Attempts to orally administer BDNF have so far yielded poor results due to the fact that BDNF is a moderately sized and charged protein and its transport through the intestinal barrier and BBB is not clear [47].

The key idea of this work is to use a low dose BDNF solution to avoid the possible side effects of current therapies (such as sensitization and allergic reactions), supporting with experimental data a new potential therapeutic approach to treat or prevent neurodegenerative diseases. The concept of low-dose is an important and innovative aspect and has been shown to be effective in many studies. For example, in vivo treatment experiments with low-dose interferon were performed in many animal species [48]. This treatment has been shown to induce dramatic clinical improvement in models of both infectious and chronic inflammatory diseases [49].

This work demonstrated, in in vitro experimental models, that BDNF is able to cross both the intestinal and BBB barrier, thus demonstrating the safety of its use.

This study also demonstrates for the first time the efficacy of low-dose BDNF SKA in counteracting oxidative damage, which is one of the mechanisms underlying age-related neurodegeneration. The possibility of administering BDNF in very small amounts is a great advantage, both for the low risk of adverse effects and for the lower cost of treatment.

In the design of the study, it was decided to use a particular solution preparation technique, which is called SKA. It has been hypothesized that the mechanism of bioactive molecules, such as hormones, neuropeptides and growth factors, subjected to SKA and administered at low dosage, consists in the sensitization or activation of some cellular (or plasma) receptor units by virtue of their high dilution, and practically in their physiological working in the order of micrograms for hormones [50] and picograms for the other messenger molecules [51]. The SKA method has been used in previous works, which showed that SKA solutions have better biological effects than corresponding solutions that did not receive the same treatment [20,21,29]. In the present study, it has been shown that BDNF SKA does not induce neuronal stress and it is able to counteract the formation of ROS.

During brain aging, the cells that show the first signs of degeneration are the astrocytes, despite the fact that subsequently the most important site of damage is represented by cortical neurons [52]. BDNF SKA is able to increase cell viability in both neuronal cells and astrocytes, representing an important resource for the health of the nervous system.

Furthermore, the aim of this study was to explore the timing of administration, to see if it was possible to identify an effective protocol that could be used in humans in the future. It can be hypothesized that the six-day protocol, consisting of a single administration followed by six days of measurements, stimulates cells without overloading normal physiological regulation. In this way a greater crossing of BDNF is achieved through the BBB, also inducing a low concentration of ROS and a reduced neuronal stress. In this way the normal cellular physiological processes are activated. The in vivo part of this research has allowed us to demonstrate that BDNF SKA has a high capacity to cross the enterohepatic circle and is able to remain in the bloodstream for at least 24 h. This makes the BDNF SKA a potential candidate for use as a food supplement. Since BDNF, necessary for the survival of neurons, is synthesized after the encoding of its specific gene, its expression was studied in this research. We observed that BDNF SKA at 24 h plus 24 h was able to induce the expression of endogenous BDNF, indicating a better effect of stimulation and induction of endogenous BDNF production. The beneficial effects of BDNF SKA have also been confirmed by the analysis of the amyloid protein precursor. Taken together, our results suggest that the simultaneous activation of at least ERK/MAPK is necessary to mediate a complete BDNF-dependent activation of the APP promoter [53].

APP protein plays a central role in the development of Alzheimer’s disease; its expression, metabolism, splicing and secretion have been demonstrated to be regulated by ligands of the membrane tyrosine kinase receptors like BDNF [54]. Furthermore, the study of the intracellular pathways demonstrated a significant increase in the expression of ApoE, which is a member of the low-density lipoprotein receptor gene family, mainly produced by the astrocytes in the brain. ApoE has been identified as the receptor that mediates amyloid β (Aβ) uptake and clearance by astrocytes, thus increasing glial LDLR levels, which may promote Aβ degradation within the brain [55,56,57]; these data indicate a positive effect on brain trophism exerted by BDNF SKA and an increase in SIRT1 phosphorylation, confirming a potential role in counteracting the known mechanisms that lead to brain aging. Indeed, SIRT1 has recently been shown to play a role in normal cognitive function and synaptic plasticity, counteracting cognitive decline and neurodegenerative disease in aging [58,59]. The effectiveness of BDNF SKA has been carefully observed, allowing the serum BDNF levels to be maintained for up to six days after a single administration; these data suggest that BDNF SKA is able to remain in the nervous tissue for a long time even in the absence of treatment, triggering its own physiological production by cells better than high-dose BDNF. All our results support the hypothesis that treatment with BDNF SKA can protect the brain over time by inducing a physiological mechanism capable of slowing cell degeneration and may be a possible therapeutic strategy for the elderly population, in order to improve cognitive function. BDNF is also able to exert effects outside the nervous system, for example on the immune system [60]. Therefore, interactions between the immune system and the effects described in this work could be hypothesized. The lack of this part could be a weak point in our research, which will be filled in subsequent research. On the other hand, one of the strengths of this study is the confirmation of the efficacy and safety of low-dose BDNF. The low-dose administration reduces the side effects of active molecules, without reducing their effectiveness is an important and innovative aspect. Indeed, the common clinical practice has long understood the essential importance of small stimuli compared to strong ones, which trigger self-regulatory and self-repairing mechanisms in the organism [61]. In brain aging there is a decline in normal antioxidant defense mechanisms leading to increased brain vulnerability and finally to the deleterious effects of oxidative damage [8]. Indeed, a large body of experimental research indicates that the brain is very susceptible to oxidative damage due to a high concentration of polyunsaturated fatty acids and transition metals that are involved in the generation of the hydroxyl radical [62,63]. Members of the ROS family, if not properly detoxified, start the process of oxidative damage, which can be defined as a chain reaction leading to sequential damage of all cellular components, in particular of lipids and proteins [64]. Oxidative stress is considered one of the main mechanisms of cellular aging due to the ease of amplification of the damage and to the large number of target molecules [8]. Cognitive deficits are the most common consequences of the aging process and they are characterized by massive neuronal loss, cognitive dysfunction and memory loss. Their incidence and prevalence continuously increase with advancing age [65]. Indeed, it has been shown that low serum BDNF levels are linked to increased cognitive impairment [66]. Moreover, BDNF helps to protect neurons from damage caused by infection or injury [45] and participates in neuronal growth and maintenance and in different aspects of activity-dependent synaptic physiology by acting across different spatial and temporal domains [67]. However, because of the difficulties associated with the administration of exogenous proteins into the central nervous system (CNS), it is important to consider the possibility of using endogenous sources of BDNF, for example, inducing increased glial cell activity. It is well known that glial cells increase expression of a variety of growth factors, including BDNF. In particular, in the adult brain, astrocytes are the cells responsible for maintaining neuronal and synaptic function [68].

## 5. Conclusions

In conclusion, this study demonstrates clear signs of the effectiveness of BDNF in cell models. Of course, it will be necessary, as a next step, to carry out a study on behavioral effects. If in this second phase the beneficial effects are also confirmed, it can be hypothesized that BDNF may in the future be a useful agent for modulating a damaged brain environment to improve recovery during demyelinating diseases and possibly other degenerative conditions.

## Figures and Tables

**Table 1:** The Papp values obtained on intestinal barrier model and plasmatic human absorption derived from Papp value.

Stimulations	30 min	1 h	3 h	4 h	5 h	6 h
1 pg/mL BDNF SKA	2.91 ± 0.3	4.18 ± 0.1	3.36 ± 0.2	3.27 ± 0.3	2.02 ± 0.2	1.97 ± 0.3
Saline	0.11 ± 0.1	0.29 ± 0.1	0.36 ± 0.1	0.5 ± 0.1	0.57 ± 0.1	0.6 ± 0.1
50 ng/mL BDNF	3 ± 0.3	3.98 ± 0.3	4 ± 0.3	3.2 ± 0.2	1.98 ± 0.2	1.02 ± 0.1
1 pg/mL BDNF SKA	32.75	39.8	36.5	34.85	26.6	26
Saline	<20	<20	<20	<20	<20	<20
50 ng/mL BDNF	33.2	38.15	39.8	33.2	25.8	20

Saline = saline solution. Data are expressed as means ± SD (%) of four independent experiments reproduced in triplicates.

**Figure A1:**
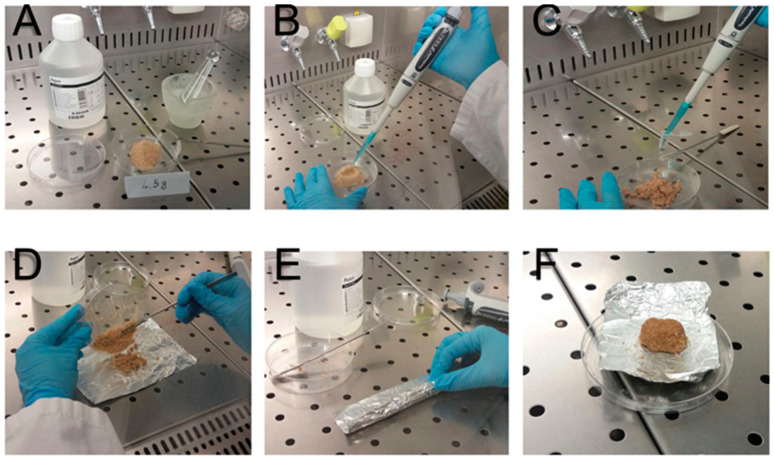
The rissoles were prepared in a sterile environment by pulverizing kibble food (Global Diet, Envigo plus) (**A**). Powder (4.5 g) of was mixed with 2.5 mL of sterile water (**B**) and 450 µl of BDNF solutions (**C**). Feed was prepared and kneaded under a hood (**D**) to form a rissole that was wrapped in a foil sterilized by heat (**E**). Soon after, the rissole was dried under the hood to maintain sterility for 18–24 h. At the end, the rissole (**F**) was placed in a cage with the mouse, which quickly ate it.
